# Insight into the evolution and functional characteristics of the pan‐genome assembly from sesame landraces and modern cultivars

**DOI:** 10.1111/pbi.13022

**Published:** 2018-12-08

**Authors:** Jingyin Yu, Agnieszka A. Golicz, Kun Lu, Komivi Dossa, Yanxin Zhang, Jinfeng Chen, Linhai Wang, Jun You, Dingding Fan, David Edwards, Xiurong Zhang

**Affiliations:** ^1^ Key Laboratory of Biology and Genetic Improvement of Oil Crops Ministry of Agriculture Oil Crops Research Institute The Chinese Academy of Agricultural Sciences Wuhan China; ^2^ Plant Molecular Biology and Biotechnology Laboratory Faculty of Veterinary and Agricultural Sciences University of Melbourne Parkville Melbourne Vic Australia; ^3^ College of Agronomy and Biotechnology, and Academy of Agricultural Sciences Southwest University Beibei Chongqing China; ^4^ Centre d'Etudes Régional pour l'Amélioration de l'Adaptation à la Sécheresse (CERAAS) Thiès Senegal; ^5^ Department of Plant Pathology & Microbiology University of California Riverside CA USA; ^6^ Sino Genomics Institute Guangdong China; ^7^ School of Biological Sciences and Institute of Agriculture University of Western Australia Perth WA Australia

**Keywords:** sesame, pan‐genome, phylogeny, positive selection, fast evolution

## Abstract

Sesame (*Sesamum indicum* L.) is an important oil crop renowned for its high oil content and quality. Recently, genome assemblies for five sesame varieties including two landraces (*S. indicum* cv. Baizhima and Mishuozhima) and three modern cultivars (*S. indicum* var. Zhongzhi13, Yuzhi11 and Swetha), have become available providing a rich resource for comparative genomic analyses and gene discovery. Here, we employed a reference‐assisted assembly approach to improve the draft assemblies of four of the sesame varieties. We then constructed a sesame pan‐genome of 554.05 Mb. The pan‐genome contained 26 472 orthologous gene clusters; 15 409 (58.21%) of them were core (present across all five sesame genomes), whereas the remaining 41.79% (11 063) clusters and the 15 890 variety‐specific genes were dispensable. Comparisons between varieties suggest that modern cultivars from China and India display significant genomic variation. The gene families unique to the sesame modern cultivars contain genes mainly related to yield and quality, while those unique to the landraces contain genes involved in environmental adaptation. Comparative evolutionary analysis indicates that several genes involved in plant‐pathogen interaction and lipid metabolism are under positive selection, which may be associated with sesame environmental adaption and selection for high seed oil content. This study of the sesame pan‐genome provides insights into the evolution and genomic characteristics of this important oilseed and constitutes a resource for further sesame crop improvement.

## Background

Sesame has been cultivated for more than 5000 years, but has been mostly restricted to the developing and emerging countries (Anastasi *et al*., 2017). Recent studies focused on the nutraceutical, pharmaceutical, cosmeceutical, industrial and ethnobotanical properties of bioactive components in sesame seeds, which renewed interest in this relatively under‐explored crop plant (Anilakumar *et al*., 2010; Cheng *et al*., 2006; Dossa *et al*., 2017a; Kanu *et al*., 2007). Cultivated sesame (*Sesamum indicum* L., 2*n* = 26) displays extensive morphological and developmental diversity including differences in branching type, plant height, flowering time, corolla colour, capsule length, number of capsule per axil, capsule edge number, seed coat colour and seed size. Recent studies revealed variation in sesame seed composition (Dossa *et al*., 2017b; Pathak *et al*., 2014; Spandana *et al*., 2013; Wang *et al*., 2012a). Sesame has a wide geographic distribution, but mainly grown in both Asia and Africa (Kobayashi, 1981; Pham *et al*., 2011). In contrast to many other crop species, cultivated sesame varieties display a high degree of genetic diversity which can be utilized for crop improvement (Dossa *et al*., 2016; Uncu *et al*., 2015; Wang *et al*., 2014a; Wei *et al*., 2014; Zhang *et al*., 2012). The genetic and associated phenotypic variation of sesame may be a result of adaptation to diverse growth habitats (Bedigian and Harlan, 1986), as well as the artificial selection pressures resulting in its partially domesticated status (Wei *et al*., 2015).

While sesame is still considered an ‘orphan crop’ with limited genomic resources, it has garnered increased interest from the scientific community, especially since the draft genome sequence has become available (Dossa *et al*., 2017a). Wang *et al*. (2014a,b) pioneered sesame genomic research with the sequencing and assembly of the modern Chinese cultivar Zhongzhi13. Sesame has a small diploid genome (∼357 Mb) and the draft assembly consisted of 274 Mb in 16 linkage groups and contained 27 148 predicted protein‐coding genes (Wang *et al*., 2014b). This reference genome was recently updated, resulting in 13 pseudomolecules encompassing 94.3% of the estimated genome size and 97.2% of the expected gene content (Wang *et al*., 2016). In addition to Zhongzhi13, four high‐quality draft genome assemblies corresponding to different genotypes representing wide geographical origins, phenotypic variation, and breeding status have also been produced. Wei *et al*. (2015) produced draft genome assemblies for two landraces Baizhima and Mishuozhima originating from Hainan and Zhejiang provinces in China. The Sesame Genome Working Group produced a 293.7 Mb draft assembly representing a modern cultivar, Yuzhi11 (Zhang *et al*., 2013), while the genome assembly of Swetha, an elite modern cultivar from India, was produced by a team from the National Bureau of Plant Genetic Resources, resulting in the largest assembly to date of 340 Mb (Kitts *et al*., 2016).

The available genome sequences representing two landraces and three modern cultivars provide valuable resources for comparative genomics and gene discovery. However, the assemblies vary in size and the number of predicted protein‐coding genes, most likely due to differences in the assembly approach and gene prediction methods, as well as the true biological variation found within the species (Bayer *et al*., 2017). A genome of a single individual is insufficient to represent the gene diversity within a species due to presence/absence and copy number variation, and a pan‐genome is required to understand the extent of the existing genomic variation (Golicz *et al*., 2016a). Within the species, genes that are present in all the individuals are considered core, while those that are present in only a subset of individuals are classed as variable or dispensable, and the union of the core and the variable genes constitutes the pan‐genome (Tettelin *et al*., 2005). Capturing the genomic diversity in a species is particularly relevant to the understanding of the phenotypic variation observed and uncovering of the underlying genes. The pan‐genome concept has been increasingly adopted and applied to higher organisms including maize (Hirsch *et al*., 2014), soybean (Li *et al*., 2014), Chinese cabbage (Lin *et al*., 2014), cabbage (Golicz *et al*., 2016b), rice (Schatz *et al*., 2014; Sun *et al*., 2017), wheat (Montenegro *et al*., 2017), Medicago (Zhou *et al*., 2017) and rapeseed (Bayer *et al*., 2017; Hurgobin *et al*., 2017).

This study uses a comparative genomic approach to analyse the five sesame genome assemblies. These were initially re‐annotated to provide a uniform framework for comparison. They were then used to construct the first sesame pan‐genome, containing 26 472 orthologous gene clusters (58.21% of the genes clusters were core and 41.79% dispensable) and 15 890 variety‐specific dispensable genes. The results obtained allowed reconstruction of the history of sesame domestication and investigation of the gene families likely contributing to agronomic traits.

## Results and discussion

### Reference‐assisted assemblies

The genomes of five sesame varieties (landraces: *S. indicum* cv. Baizhima and Mishuozhima, and modern cultivars: *S. indicum* var. Zhongzhi13, Yuzhi11 and Swetha) found in different geographical areas (Hainan, Zhejiang, Hubei, and Henan provinces of China, and India) have been sequenced and assembled (Kitts *et al*., 2016; Wang *et al*., 2014b; Wei *et al*., 2015; Zhang *et al*., 2013) (Table 1 and Figure S1). The available genome sequence of Zhongzhi13 has been assembled to the pseudomolecule level, whereas the genome sequences of Baizhima, Mishuozhima, Yuzhi11 and Swetha are available as contigs and scaffolds. The available assemblies range in size from 210.76 Mb for Yuzhi11 to 340.46 Mb for Swetha.

**Table 1:** Sample information, assembly and annotation for five sesame varieties

Categories	Zhongzhi13	Yuzhi11	Swetha	Baizhima	Mishuozhima
Location	Hubei, China	Henan, China	New Delhi, India	Hainan, China	Zhejiang, China
Assembly type	Chromosome	Scaffold	Scaffold	Contig	Contig
Total length (bp)	272 734 981	210 758 237	340 463 922	266 768 502	253 855 660
Original					
Contig N50 (bp)	53 067	17 903	8725	47 354	47 930
Scaffold N50 (bp)	20 257 639	324 903	22 222	47 354	47 930
Improved scaffold N50 (bp)	–	12 372 216	23 861 055	16 359 355	16 328 919
Predicted gene numbers	40 219	29 601	49 428	37 083	36 410
Refined gene numbers	36 189	26 022	41 859	31 558	30 995

In order to facilitate comparisons between varieties, Chromosomer v 0.1.4a was used to align available contigs and scaffolds to the Zhongzhi13 reference genome and build chromosome‐level assemblies for the four sesame varieties (Baizhima, Mishuozhima,Yuzhi11 and Swetha) (Tamazian *et al*., 2016). After reference‐assisted scaffolding, the original scaffold N50 sizes for the four sesame varieties were improved from Kb level (ranging from 47.354 Kb for Baizhima to 324.9 Kb for Yuzhi11) to Mb level (ranging from 16.33 Mb for Baizhima to 23.86 Mb for Swetha). Approximately 81.87%, 85.95%, 91.14% and 81.52% of total assembled genome sequences in Baizhima, Mishuozhima, Swetha and Yuzhi11, respectively, were anchored to the 13 chromosomes based on Zhongzhi13 genome (Table S1).

### Gene re‐annotation of five sesame varieties

**Figure 1:**
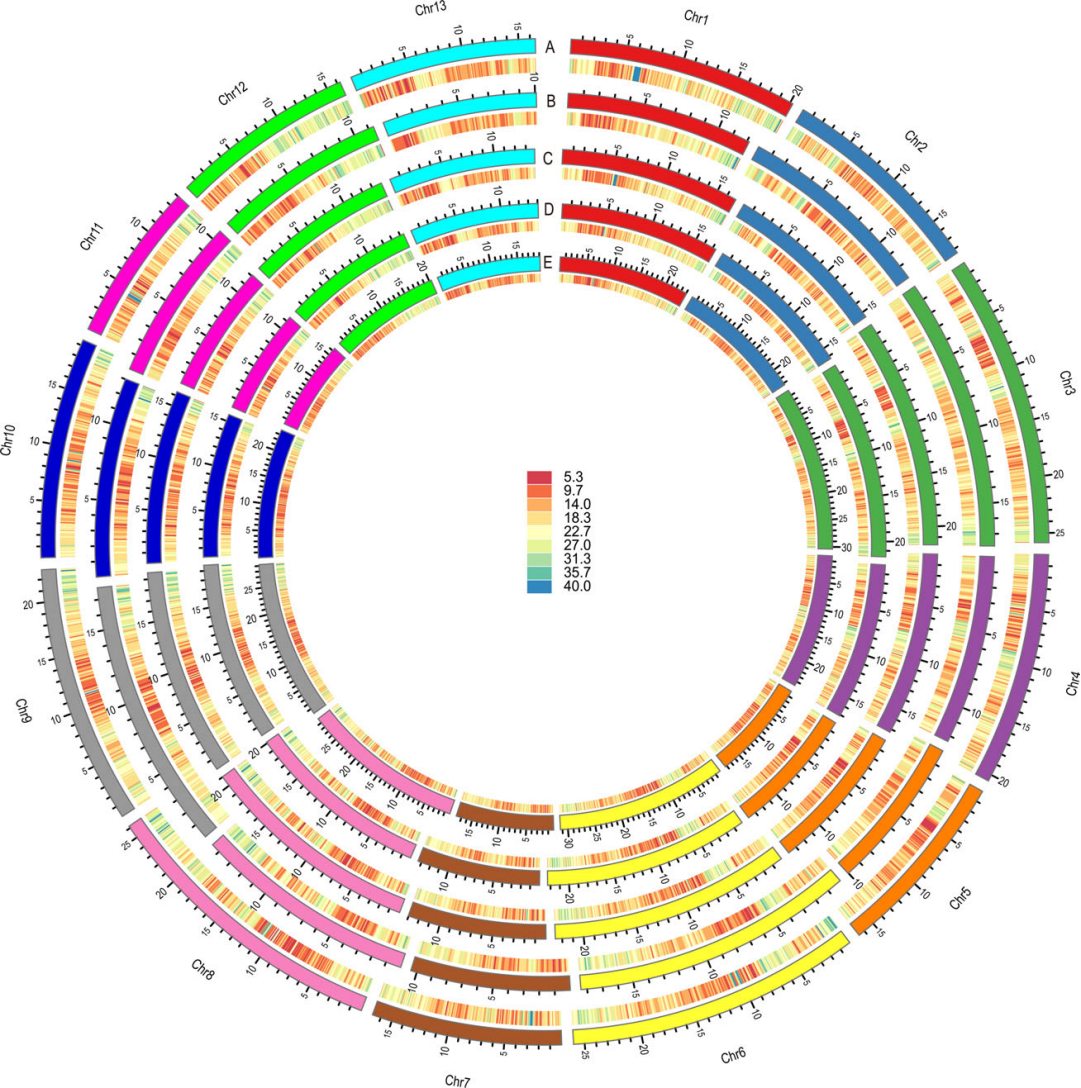
Chromosomal distribution of protein‐coding genes among five sesame varieties. The chromosomes and gene density on corresponding chromosomes among five sesame varieties are distinguished by different colours. a–e represents Zhongzhi13, Yuzhi11, Baizhima, Mishuozhima and Swetha respectively.

The Zhongzhi13 reference genome and the four newly constructed assemblies were re‐annotated using the Maker v2.31.9 annotation pipeline, which combines *ab initio* gene prediction with protein homology and transcriptomic evidence (Cantarel *et al*., 2008). We predicted 36 189, 26 022, 41 859, 31 558 and 30 995 protein‐coding genes in Zhongzhi13, Yuzhi11, Swetha, Baizhima and Mishuozhima respectively (Figure 1). The 36 189 protein‐coding genes for Zhongzhi13 represent a 33% increase over the previous report (27 148 genes) (Wang *et al*., 2014b). Comparison of the existing and the newly generated Zhongzhi13 annotations identified 12 150 genes unique to the new set, 249 genes unique to the old set with the remainder shared by the two annotations (Figure S2). The annotation statistics including gene length, transcript length and CDS length were comparable between the two annotations (Table S2). Gene ontology (GO) analysis revealed that these newly identified genes were annotated with functions related to RNA, nucleic acid and protein binding, RNA‐dependent DNA replication, ATP binding and oxidation‐reduction processes, RNA transport, endocytosis, purine metabolism, glycolysis/gluconeogenesis, and amino sugar and nucleotide sugar metabolism (Data S1). The updated annotation of the Zhongzhi13 assembly provides a new resource for the study of sesame biology and evolution. The observed variation in gene numbers for the five sesame varieties provides an opportunity to construct the pan‐genome of sesame and identify potential links between gene presence/absence variation and phenotypic diversity.

### Construction of sesame pan‐genome

**Figure 2:**
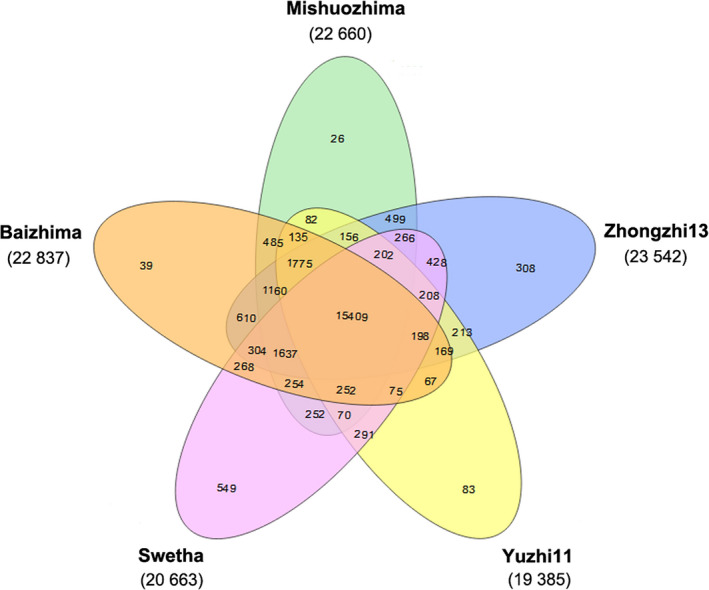
Venn diagram of the distribution of gene clusters among five sesame varieties. The numbers of gene clusters are displayed for every species. The intersections between species indicate the numbers of shared gene clusters, whereas the numbers of unique clusters are shown in species‐specific areas.

The sesame pan‐genome was constructed using whole genome alignment of the five varieties. The total pan‐genome size was 554.05 Mb, containing 258.79 Mb and 295.26 Mb of the core and the dispensable genome sequence respectively. OrthoMCL v1.4 was used to identify orthologous gene clusters representing the genic content of the five sesame genomes, as well as seven other plant species (*Utricularia gibba*,* Solanum lycopersicum*,* Solanum tuberosum*,* Vitis vinifera*,* Arabidopsis thaliana*,* Zea mays* and *Oryza sativa*). In total, 40 871 orthologous gene clusters were identified (Figure 2 and Table S3). The sesame pan‐genome was composed of 26 472 orthologous gene clusters (interpreted as corresponding to gene families) and 15 890 unclustered (or variety‐specific) genes among the five sesame genomes. Out of the total number of orthologous gene clusters in the pan‐genome, 15 409 (58.21%) are core (present across all five sesame genomes), whereas the remaining 41.79% (11 063) and 15 890 variety‐specific genes are dispensable. The relatively high proportion of dispensable orthologous gene clusters and variety‐specific genes underscores the genomic diversity of sesame. The genomic diversity could in turn contribute to the phenotypic diversity and local adaptations of sesame.

### Evolution and domestication of the different sesame varieties

**Figure 3:**
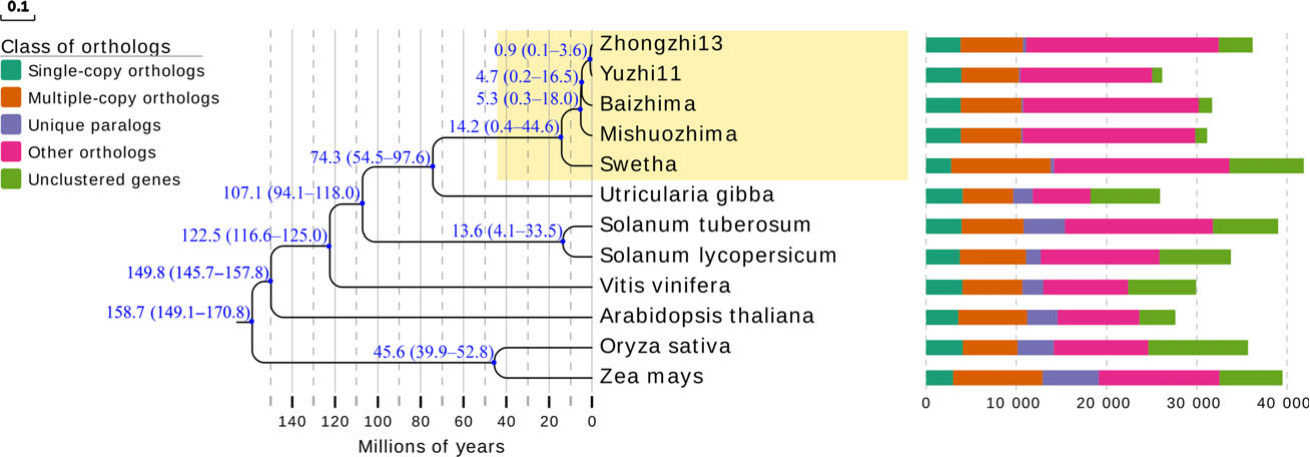
Phylogeny and distribution of different gene clusters for five sesame varieties. The numbers on the branches represent divergence time. The bars represent the numbers of different types of gene clusters among five varieties.

Using 518 199 commonly conserved sites from alignments of 1010 conserved single‐copy gene orthologous groups from 12 plants, we constructed a phylogenetic tree to examine the evolutionary relationships among the two sesame landraces and the three modern cultivars (Figure 3). We used the known divergence time between species in Timetree as calibration points to estimate the divergence time among the sesame varieties (Kumar *et al*., 2017). We estimate that *U. gibba* and the *Sesamum* lineage diverged ~66.1 MYA, which is consistent with a previous report (Unver *et al*., 2017). Swetha from India and the sesame varieties from China were estimated to have diverged ~14.2 MYA, suggesting potential high levels of genomic diversity between Indian and Chinese sesame varieties. The largest number of unique and dispensable orthologous gene clusters was detected in Swetha, which reflects its greater evolutionary distance from the other varieties. Our results also suggest that sesame landraces (Baizhima and Mishuozhima) and sesame modern cultivars (Zhongzhi13 and Yuzhi11) in China diverged ~4.7 MYA, while modern Chinese sesame cultivars (Zhongzhi13 and Yuzhi11) were estimated to have diverged ~0.9 MYA, which is consistent with the breeding history of these two modern cultivars originating from neighbouring provinces (Hubei and Henan provinces) in China. The greatest amount of shared genomic sequence was also found between the modern Chinese cultivars Zhongzhi13 and Yuzhi11.

Most genera of the Pedaliaceae family, which sesame belongs to, were grown chiefly in tropical Africa and most wild species of *Sesamum* are also found exclusively in Africa. Initially, it was believed that sesame was first domesticated in Africa. However, the evidence from genetic and chemical data suggests that the Indian subcontinent was the earliest place of sesame domestication (Bedigian, 2003). High levels of genomic diversity between the Indian and Chinese sesame varieties indicate that sesame modern cultivars from India and China might stem from independent domestication events. The analysis suggests that the Indian modern cultivar Swetha was domesticated earlier than Chinese modern cultivars, which is consistent with the previous reports that sesame was firstly domesticated in the Indian subcontinent (Bedigian, 2003).

### Origin of sesame core and dispensable genes

The 15 409 gene families of sesame core genome contained 23 372, 20 876, 27 557, 22 343 and 22 146 genes in Zhongzhi13, Yuzhi11, Swetha, Baizhima and Mishuozhima, with the sesame dispensable genome composed of 12 817, 5146, 14 302, 9215 and 8849 genes in Zhongzhi13, Yuzhi11, Swetha, Baizhima and Mishuozhima respectively (Table 2).

**Table 2:** The influences of different evolutionary events on the core and dispensable genes among different sesame varieties

Categories	Zhongzhi13	Yuzhi11	Swetha	Baizhima	Mishuozhima
Total Genes	36 189	26 022	41 859	31 558	30 995
Core genes					
Total	23 372	20 876	27 557	22 343	22 146
WGD‐type	10 932 (46.77%)	10 810 (51.78%)	9931 (36.04%)	10 221 (45.75%)	10 190 (46.01%)
TD‐type	2076 (8.88%)	1432 (6.86%)	4556 (16.53%)	1907 (8.54%)	1873 (8.46%)
Dispensable genes					
Total	12 817	5146	14 302	9215	8849
WGD‐type	898 (7.01%)	775 (15.06%)	1671 (11.68%)	789 (8.56%)	781 (8.83%)
TD‐type	1001 (7.81%)	286 (5.56%)	2323 (16.24%)	814 (8.83%)	774 (8.75%)

Sesame experienced a whole genome duplication event (WGD) approximately 71 million years ago (MYA) leading to many genes being present in two copies (Wang *et al*., 2014b). Comparison of the gene sets among different sesame varieties identified 11 830, 11 585, 11 602, 11 010 and 10 971 orthologous gene pairs in Zhongzhi13, Yuzhi11, Swetha, Baizhima and Mishuozhima compared to *V. vinifera*, which suggests that these genes in sesame were generated from a WGD (Figure S3). For protein‐coding gene sets, we found that 46.77% (10 932), 51.78% (10 810), 36.04% (9931), 45.75% (10 221) and 46.01% (10 190) of the core genes were generated by WGD event. For the dispensable genome, 7.01% (898), 15.06% (775), 11.68% (1671), 8.56% (789) and 8.83% (781) of dispensable genes found in varieties Zhongzhi13, Yuzhi11, Swetha, Baizhima and Mishuozhima respectively are influenced by WGD event (Data S2).

Tandem duplications (TD) occur more frequently and on smaller scale than WGD and lead to the expansion of gene families (Graham, 1995). Using sequence similarity analysis and position information, we identified 1309, 751, 3089, 1170 and 1134 tandem arrays covering 3077, 1718, 6879, 2721 and 2647 tandem duplicated genes in Zhongzhi13, Yuzhi11, Swetha, Baizhima and Mishuozhima respectively. We found that 8.88% (2076), 6.86% (1432), 16.53% (4556), 8.54% (1907) and 8.46% (1873) of the core genes found in Zhongzhi13, Yuzhi11, Swetha, Baizhima and Mishuozhima, respectively, originated from TD events, and for the dispensable genome, we identified 2199, 1046, 4848, 1925 and 1853 tandem duplicated genes in Zhongzhi13, Yuzhi11, Swetha, Baizhima and Mishuozhima representing 7.81%, 5.56%, 16.24%, 8.83% and 8.75% of the dispensable genes. TD analyses revealed that Swetha has a higher proportion of TD‐type genes in the core and dispensable gene set than other varieties. The genome of Swetha has undergone more TD events when compared with the Chinese sesame varieties, which may contribute to its higher genetic distance from other varieties (Data S3). The availability of additional gene copies which can undergo sequence divergence and neo‐functionalization may also contribute to potentially higher phenotypic plasticity of Swetha.

### Variation of sesame dispensable genome among landraces and modern cultivars

Pan‐genome analysis identified 11 063 gene families and 15 890 variety‐specific genes as dispensable. We investigated the partitioning of the dispensable gene set between modern cultivars (Zhongzhi13, Yuzhi11 and Swetha) and landraces (Baizhima and Mishuozhima). We detected 2080 gene families and 13 094 variety‐specific genes, which were unique to the modern cultivars, while 552 gene families and 2796 variety‐specific genes were found only in the sesame landraces (Data S4). KEGG analysis suggests that the genes unique to the modern cultivars are associated with functions involved in energy metabolism, nucleotide metabolism, cell growth and death, and amino acid metabolism; including pathways of oxidative phosphorylation (ko00190), photosynthesis (ko00195), purine metabolism (ko00230), pyrimidine metabolism (ko00240), cell cycle (ko04110) and cysteine and methionine metabolism (ko00270). The genes with functions related to energy metabolism, growth and development, as well as biomass accumulation could have contributed to the advantageous traits selected during cultivation. The analysis of landraces‐specific genes, highlighted functions related to environmental adaptation, signal transduction, protein folding, sorting and degradation, and transport and catabolism; including the pathways of plant‐pathogen interaction (ko04626), sphingolipid signalling (ko04071), PI3K‐Akt signalling (ko04151), protein processing in endoplasmic reticulum (ko04141), and phagosome (ko04145). These genes possibly reflect environmental adaptation capabilities found in sesame landraces, which may have been lost in modern cultivars due to artificial selection. The results suggest that even for an ‘orphan crop’ like sesame there are genes available in the wider species pool which are missing from the modern cultivars and may be unavailable for breeding programs. Due to presence of unique genes landraces should be considered potential donors of valuable traits.

To investigate the potential differences accumulated during artificial selection in China and India, we studied the variation of unique orthologous gene clusters and variety‐specific genes found in the Chinese and Indian modern cultivars. We identified 604 unique orthologous gene clusters and 1498 variety‐specific genes in the Chinese cultivars, which could be mapped to 220 KEGG pathways and 549 unique orthologous gene clusters and 4433 variety‐specific genes in the Indian cultivar mapped to 280 KEGG pathways (Data S5). The unique genes in the Chinese and Indian cultivars were mapped 185 common KEGG pathways (including plant hormone signal transduction and phenylpropanoid biosynthesis). The genes unique to the Chinese cultivars were annotated as involved in energy metabolism, lipid metabolism and amino acid metabolism, while the genes unique to the Indian cultivar were mainly involved in environmental adaptation, signal transduction and cellular interactions. The involvement of the genes unique to the Chinese cultivars in pathways related to seed quality (energy metabolism, lipid metabolism and amino acid metabolism) suggest that the Chinese cultivars may have undergone stronger artificial selection for seed quality related traits rather than disease resistance and environmental adaptation.

### Change of gene family size during evolution of sesame

The increase or reduction of the gene family size may be associated with important biological functions which differentiate sesame from other plant species (Lau *et al*., 2016; Lespinet *et al*., 2002). We identified 113 gene families which are expanded in sesame compared to *U. gibba*,* S. lycopersicum*,* S. tuberosum*,* V. vinifera*,* A. thaliana*,* Z. mays* and *O. sativa*. These gene families were mapped to 65 known KEGG orthologous groups and 81 KEGG pathways (Kanehisa *et al*., 2017) (Data S6). Several of the expanded gene families are involved in defense response, flavonoid biosynthesis (ko00941) and lipid biosynthesis.

Pathogen resistance is one of the major factors behind crop productivity. We have identified two expanded gene families, containing orthologues of important defense response and stress tolerance genes RPM1 and FRY1 (Grant *et al*., 1995; Hsu *et al*., 2013; Robatzek and Somssich, 2002). RPM1 is a resistance (R) protein that specifically recognizes a bacterial avirulence protein, resulting in effector‐triggered immunity (Gassmann and Bhattacharjee, 2012). The R genes are known to be subject to presence/absence, copy number and resulting gene family size variation (Lespinet *et al*., 2002; Richter and Ronald, 2000). The expansion of RPM1 might improve sesame resistance to bacterial pathogens. FRY1 is a regulator of abscisic acid and stress signalling in *A. thaliana*. Expansion of FRY1 gene family may result in increased freezing, drought and salt‐stress tolerance (Xiong *et al*., 2001).

Flavonoids are a major class of plant secondary metabolites, which are involved in multiple biological functions including abiotic stress tolerance and protection against UV‐B radiation (Falcone‐Ferreyra *et al*., 2012). The flavonol synthase (FLS), flavonoid 3′‐monooxygenase, shikimate O‐hydroxycinnamoyltransferase (HCT) and leucoanthocyanidin reductase (LAR) show evidence of genes family expansion (Table 3) and play important roles in flavonoid biosynthesis. In maize, which contains two copies of the FLS gene, both genes appear functional and show evidence of expression, especially under light stress (Falcone‐Ferreyra *et al*., 2012). The expansion of the FLS, HCT and LAR gene families might promote flavonoid biosynthesis and accumulation in sesame and promote increased abiotic stress tolerance.

**Table 3:** Statistics of expansion and contraction of gene families in five sesame varieties compared to *U. gibba*,* S. lycopersicum*,* S. tuberosum*,* V. vinifera*,* A. thaliana*,* Z. mays* and *O. sativa*

Family	Total genes	Sin					Ugi	Sly	Stu	Vvi	Ath	Zma	Osa	Description
		Zhongzhi13	Yuzhi11	Swetha	Baizhima	Mishuozhima								
F3′H	71	9	5	8	6	7	0	7	9	4	0	11	5	Flavonoid 3′‐monooxygenase
HCT	28	5	4	3	5	5	1	1	2	1	1	0	0	Shikimate O‐hydroxycinnamoyltransferase
FLS	28	7	2	6	3	3	1	1	1	3	0	0	1	Flavonol synthase
LAR	20	4	0	6	4	4	0	0	0	2	0	0	0	Leucoanthocyanidin reductase
BGLU	223	33	18	29	27	31	7	8	8	17	16	16	13	Beta‐glucosidase
CCR	36	5	4	5	5	5	1	3	0	4	2	1	1	Cinnamoyl‐CoA reductase
WSD1	43	1	1	1	1	1	12	3	3	10	4	2	4	O‐acyltransferase

Oil and fatty acid content of sesame seeds are an important research focus area. Four gene families involved in lipid metabolism showed expansion during evolution of sesame (CYP1A1 and CYP1B1 involved in steroid hormone biosynthesis; glycerol‐3‐phosphate acyltransferase (GPAT) involved in glycerophospholipid and glycerolipid metabolism; linoleate 9S‐lipoxygenase (LOX1_5) involved in linoleic acid metabolism). The expansion could strengthen the biosynthesis of steroid hormones and the metabolism of glycerophospholipids, glycerolipids and linoleic acids in sesame, promoting the accumulation of oil and fatty acid content in sesame.

Compared with *U. gibba*,* S. lycopersicum*,* S. tuberosum*,* V. vinifera*,* A. thaliana*,* Z. mays* and *O. sativa*, 21 families with reduced gene number were observed in sesame. Functional annotation of these gene families indicates roles in the pathways of cutin, suberin and wax biosynthesis (ko00073) and spliceosome (ko03040). For cutin, suberin and wax biosynthesis, the wax‐ester synthase/diacylglycerol O‐acyltransferase 1 (WSD1) gene family was identified as reduced in size in sesame compared with other plant species. WSD1 is an important enzyme involved in cutin, suberin and wax biosynthesis (King *et al*., 2007). Wax esters are neutral lipids, which are composed of aliphatic alcohols and acids. In plants, they mostly exist in the cuticle of primary shoot surfaces and also accumulate with high concentrations in the seed oils of oil crops (Li *et al*., 2008). The reduction of number of WSD1 genes in sesame could affect cuticle and seed composition.

The analysis suggests possible roles of gene family expansion in disease resistance, flavonoid biosynthesis and lipid metabolism. While the changes in the gene family size point to modifications of corresponding pathways and the resulting rate of metabolite production/accumulation, the interplay between the abundance and activity rates of all the enzymes, including the rate‐limiting enzymes, involved will determine the ultimate end‐product concentration.

### Positively selected and fast‐evolving genes in sesame

**Figure 4:**
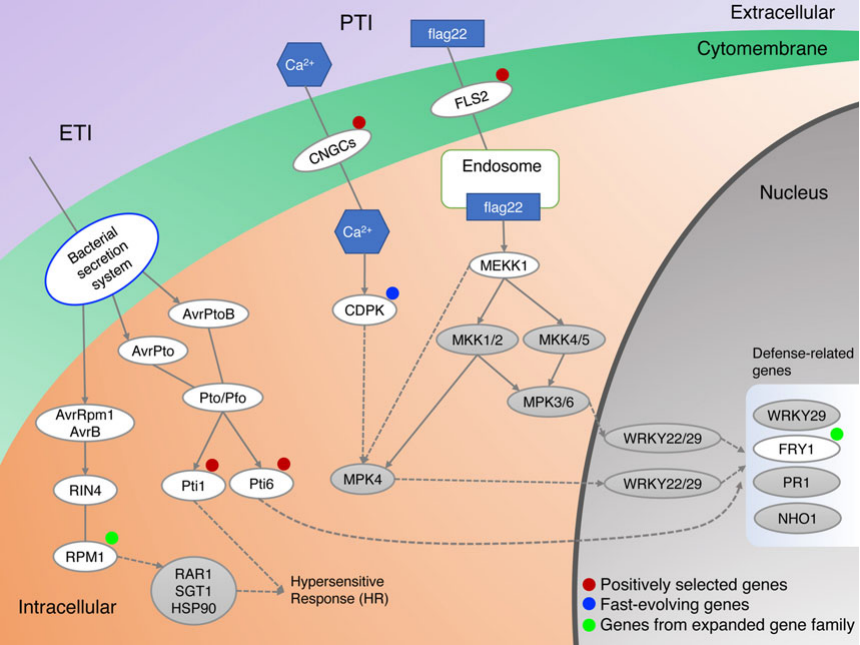
Fast‐evolving and positively selected genes, as well as genes from expended gene families in plant‐pathogen interaction pathway. Plants have evolved an immunity system with multiple‐layers of protection against invading pathogens including pathogen‐associated molecular pattern triggered immunity (PTI) and effector‐triggered immunity (ETI) (Boller and He, 2009; Zipfel, 2008). The CNGC and FLS2 play an important role in the PTI, while the Pti1 and Pti6 are critical immune genes in ETI (Chinchilla *et al*., 2006; Gu *et al*., 2002; Jia *et al*., 1997; Ma *et al*., 2009; Zhou *et al*., 1995, 1997). CNGC and FLS2 are involved in signal perception (Chinchilla *et al*., 2006; Ma *et al*., 2009), while Pti‐1 and Pti‐6 and CPK act in downstream signalling and transcriptional cascades (Gu *et al*., 2002; Jia *et al*., 1997; Zhou *et al*., 1995, 1997). The calcium‐dependent protein kinase (CPK) is involved in abscisic acid‐activated signalling pathway, intracellular signal transduction and plant immunity (Harmon *et al*., 2000; Kadota *et al*., 2015). RPM1 specifically recognizes the AvrRpm1 type III effector avirulence protein and triggers defense responses including the hypersensitive response (Grant *et al*., 1995). Red solid circles represent positively selected genes, blue solid circle represent fast‐evolving genes, and cyan‐blue solid circles represent the genes from expended gene families.

**Figure 5:**
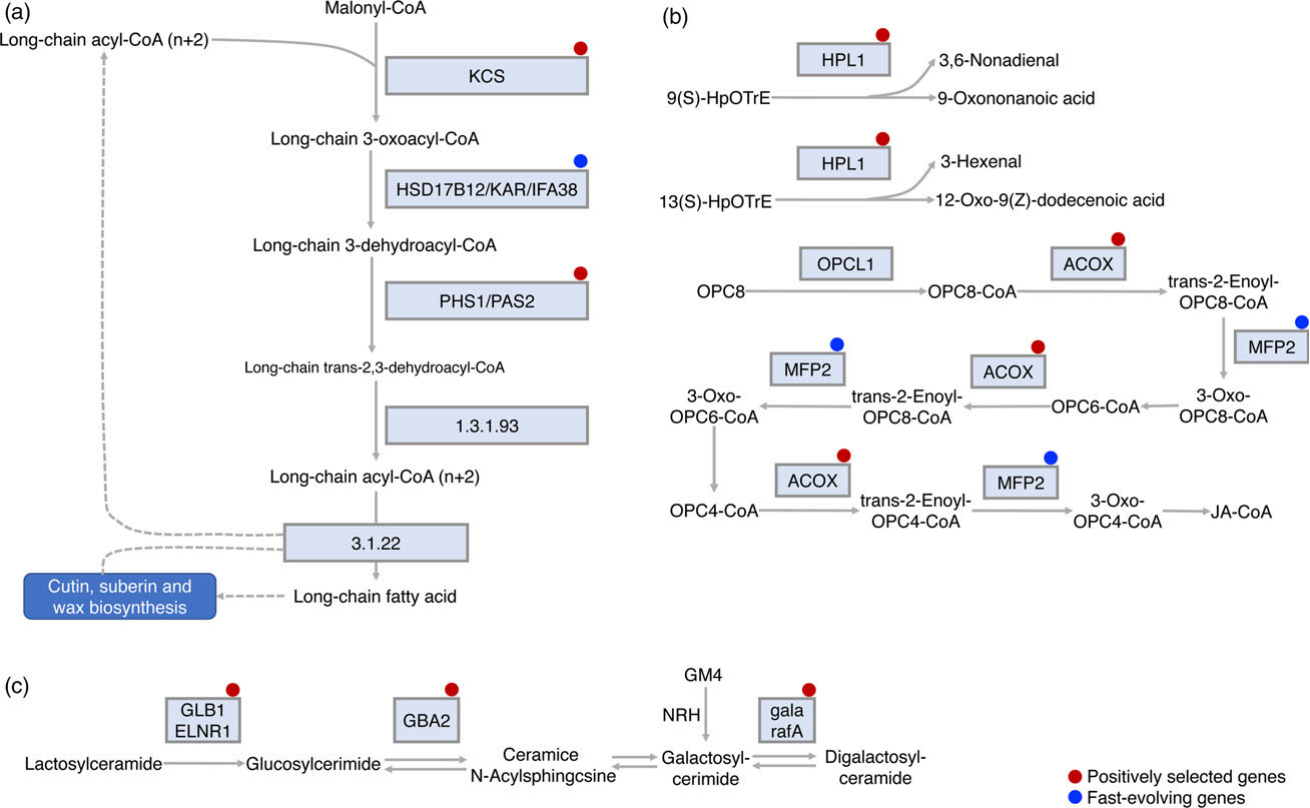
Fast‐evolving and positively selected genes in lipid metabolism. (a) Fatty acid elongation. Very‐long‐chain fatty acids (VLCFAs) are synthesized via four successive enzymatic reactions including condensation, reduction, dehydration, and a second reduction (Beaudoin *et al*., 2009; Denic and Weissman, 2007). PHS1 and KAR are also involved unsaturated fatty acids biosynthesis. Another enzyme, ACOX1 is a rate‐limiting enzyme in peroxisomal fatty acids β‐oxidation (Oaxaca‐Castillo *et al*., 2007). (b) Alpha‐Linolenic acid metabolism. Alpha‐Linolenic acid is a precursor compound and plays an important role in human health. (c) Sphingolipid metabolism. Sphingolipids and corresponding metabolites are not only key elements of cellular membranes, but are also involved in signal transduction for example in cell growth, differentiation, senescence, and programmed cell death. E3.2.1.22B (or gala or rafA) is involved in carbohydrate metabolic process and cell wall organization (Tapernoux‐Luthi *et al*., 2004). GLB1 (ELNR1) is a glycosidase, which catalyzes the hydrolysis of terminal β‐linked galactose residues (Ohto *et al*., 2012). GBA2 plays a role in glucosylceramide metabolism (Boot *et al*., 2007). Red and blue solid circles represent positively selected genes and fast‐evolving genes respectively.

Using the inferred phylogenetic relationships between the sesame varieties and five other species (*U. gibba*,* S. lycopersicum*,* S. tuberosum*,* V. vinifera*, and *A. thaliana*), we searched for genes which show evidence of positive selection or are fast evolving in sesame. Using the branch model, we found 173 candidate genes that are evolving significantly faster in sesame ancient branch compared with the remaining branches (Data S7). Using branch‐site model, we detected a set of 212 candidate genes that showed positive selection in sesame ancient branch compared with other branches. Through comparative analysis of positively selected and fast‐evolving genes, we obtained 27 genes that were fast‐evolving and contained positively selected sites in sesame. Orthologues of four genes encoding proteins involved in plant‐pathogen interaction: cyclic nucleotide gated channel (CNGC), Flagellin sensitive 2 (FLS2), Pto‐interacting 1 (Pti1) and Pto‐interacting 6 (Pti6) showed evidence of positive selection in sesame (Figure 4). Those genes could contribute to enhanced disease resistance. We have also identified 12 positively selected genes and seven fast‐evolving genes in lipid metabolism in sesame, which could be mapped to ten KEGG lipid metabolism pathways (Table 4). The genes were involved in fatty acid elongation and biosynthesis of unsaturated fatty acids (Figure 5a), alpha‐linolenic acid metabolism (Figure 5b) and sphingolipid metabolism (Figure 5c). The genes identified could promote changes in lipid metabolism which differentiate sesame from other plant species. Together the analysis of gene family expansion and gene positive selection/fast evolution give insights into the biochemical pathways which have been altered during sesame evolution.

**Table 4:** Statistics of positively selected and fast‐evolving genes in lipid metabolism in sesame

Pathway ID	Description	Numbers of positive selected genes	Numbers of fast‐evolving genes
ko00062	Fatty acid elongation	2 (KAS, PHS1 or PAS2)	1 (HSD17B12 or KAR or IFA38)
ko00071	Fatty acid degradation	1 (E1.3.3.6 or ACOX1 or ACOX3)	1 (MFP2)
ko00073	Cutin, suberin and wax biosynthesis	0	1 (CYP86B1)
ko00140	Steroid hormone biosynthesis	0	1 (HSD17B12 or KAR or IFA38)
ko00561	Glycerolipid metabolism	1 (E3.2.1.22B or galA or rafA)	0
ko00564	Glycerophospholipid metabolism	0	1 (PCYT2)
ko00565	Ether lipid metabolism	1 (PLA2G7 or PAFAH)	0
ko00592	Alpha‐Linolenic acid metabolism	2 (HPL1, E1.3.3.6 or ACOX1 or ACOX3)	1 (MFP2)
ko00600	Sphingolipid metabolism	3 (E3.2.1.22B or galA or rafA, GLB1 or ELNR1 or srfJ, GBA2)	0
ko01040	Biosynthesis of unsaturated fatty acids	2 (PHS1 or PAS2, E1.3.3.6 or ACOX1 or ACOX3)	1 (HSD17B12 or KAR or IFA38)

## Conclusions

In summary, the improved genome assemblies and annotations of the sesame landraces and cultivars provide extensive genomic resources for studying biology, genome diversity and evolution of sesame. Phylogenetic analysis revealed that sesame modern cultivar Swetha and other four sesame varieties from China grouped into different clusters, suggesting independent domestication events. The analysis of the sesame pan‐genome provided novel insights into the expansion and contraction of gene families, the size and origin of the sesame core and dispensable genomes, as well as the functional difference between landraces and cultivars. Comparative evolutionary analysis revealed that the fast‐evolving and positively selected genes which participate in plant‐pathogen interaction and lipid metabolism could be responsible for improved environmental adaption and promotion of high accumulation of oil and fatty acid in sesame seeds.

## Materials and methods

### Data resources

The assembled genome sequences of *S. indicum* var. Zhongzhi13, Yuzhi11 and Swetha, *S. indicum* cv. Baizhima and Mishuozhima were downloaded from http://ocri-genomics.org/Sinbase_v2.0 (Wang *et al*., 2016), https://www.ncbi.nlm.nih.gov/Traces/wgs/?val=MBSK01 (Kitts *et al*., 2016), https://www.ncbi.nlm.nih.gov/Traces/wgs/?val=JPLX01 (Kitts *et al*., 2016), and https://www.ebi.ac.uk/ena/data/view/ (Wei *et al*., 2015) respectively. The genome data of *Utricularia gibba* PLAZA_v4 were downloaded from https://bioinformatics.psb.ugent.be (Ibarra‐Laclette *et al*., 2013). The genome data of *Solanum lycopersicum* SL2.50, *Solanum tuberosum* SolTub_3.0, *Vitis vinifera* IGGP_12x, *Arabidopsis thaliana* TAIR10, *Zea mays* AGPv4 and *Oryza sativa* IRGSP‐1.0 were downloaded from Ensembl Genomes Release 37 (http://ensemblgenomes.org/) (Kersey *et al*., 2018).

### Chromosome‐assisted assembly

Chromosomer v 0.1.4a (Tamazian *et al*., 2016) was used to construct chromosome‐level assemblies of *S. indicum* var. Yuzhi11, *S. indicum* var. Swetha, *S. indicum* cv. Baizhima, and *S. indicum* var. Mishuozhima from contigs and scaffolds using their alignments to reference genome of *S. indicum* var. Zhongzhi13. First, the scaffold or contig genomic sequences of the four sesame varieties were aligned to the reference genome using BLASTN v2.2.30 (‐*E* 1e‐30 and ‐*m* 8) (Altschul *et al*., 1997). The results of BLASTN alignments were passed to Chromosomer to connect the mapping fragments with 100 N linkers (fragmentmap ‐*r* 1.05) and anchor them to the reference genome chromosomes. The unplaced fragments were also collected and added to the anchored contigs/scaffolds to produce the final assemblies of four sesame varieties.

### Gene prediction and annotation

Maker (2.31.9) annotation pipeline was used to re‐annotate the genomes of the five sesame varieties (Cantarel *et al*., 2008). Protein‐coding genes from *U. gibba* PLAZA_v4, *S. lycopersicum* SL2.50, *S. tuberosum* SolTub_3.0, *V. vinifera* IGGP_12x and *A. thaliana* TAIR10, and 44,905 ESTs download from NCBI dbEST (12.26.2017) were used as homology evidence. *Ab initio* gene prediction was performed with Augustus (v2.7) and Fgenesh (from MOLQUEST 2.4.5) (Hoff *et al*., 2016; Victor Solovyev *et al*., 2018). Based on the comparison of annotation results from *ab initio* prediction, protein homology evidence and transcriptomic evidence, we selected the genes with 50% of the coding regions supported by protein homology and/or transcriptomic evidence for further analysis. We removed the newly identified genes supported solely by *ab initio* prediction from one source (Augustus v2.7 or Fgenesh). We removed the fragmented genes (two genes supported by only one homologous gene from other species), and we used Exonerate to supplement the removed gene in same genomic location. The predicted protein‐coding genes were annotated by comparisons Gene Ontology (Ashburner *et al*., 2000), UniProtKB/Swiss‐Prot (O'Donovan *et al*., 2002) and KEGG Release 85.1 databases (Kanehisa *et al*., 2017), and by using InterPro 63.0 by searching member databases, including Pfam v31.0, Gene3D v 4.1.0, CDD v3.14, Hamap v201701.18, Phobius v1.01, Pirsf v3.02, Prints v42.0, Prodom v2006.1, Prosite v20.132, Smart v7.1, Superfamily v1.75 and Tigrfam v15.0, with default parameters (Quevillon *et al*., 2005).

### Construction of the sesame pan‐genome

Mugsy v1.2.3 was employed to detect alignments among *S. indicum* var. Zhongzhi13, *S. indicum* var. Yuzhi11, *S. indicum* var. Swetha, *S. indicum* cv. Baizhima, and *S. indicum* cv. Mishuozhima using the default parameters (Angiuoli and Salzberg, 2011). Based on genomic alignments, the regions shared by five sesame varieties, were defined as the sesame core genome, and the regions shared by some varieties were defined as the dispensable genome. The sesame core and dispensable genomes constitute the sesame pan‐genome.

### Gene clustering

The protein‐coding genes from *S*. *indicum* var. Zhongzhi13, Yuzhi11 and Swetha, *S. indicum* cv. Baizhima and Mishuozhima, *U. gibba* PLAZA_v4, *S. lycopersicum* SL2.50, *S. tuberosum* SolTub_3.0, *V. vinifera* IGGP_12x, *A. thaliana* TAIR10, *Z. mays* AGPv4 and *O. sativa* IRGSP‐1.0 were downloaded from species‐specific and public databases for gene clustering analysis. All protein sequences were compared using all‐by‐all BLASTP v2.2.30 search (‐*E* value 1e‐05). OrthoMCL v1.4 was used to cluster genes into orthologous gene families with default parameters (Li *et al*., 2003). The gene families were used to estimate the sesame pan‐genome size. The gene families shared by the five sesame varieties constitute the core gene sets, while the gene families shared by less than five sesame varieties and variety‐specific genes constitute the dispensable gene set.

### Gene family expansion and contraction

CAFÉ v2.2 was used to detect gene family expansion and contraction (using divergence time instead of branch length). Sequences representing the eight species (of *S. indicum* var. Zhongzhi13, Yuzhi11 and Swetha, *S. indicum* cv. Baizhima and Mishuozhima, *U. gibba* PLAZA_v4, *S. lycopersicum* SL2.50, *S. tuberosum* SolTub_3.0, *V. vinifera* IGGP_12x, *A. thaliana* TAIR10, *Z. mays* AGPv4 and *O. sativa* IRGSP‐1.0) were used in the analysis (De Bie *et al*., 2006).

### Identification of WGD‐ and TD‐type genes

Sesame has experienced a WGD event leading to the duplication of its genomic and genic content. We employed the MCscanX (11.13.2012) package to identify orthologous gene pairs within the syntenic regions between sesame and grape genomes (*e* = 1e‐20, *u* = 1 and *s* = 15) (Wang *et al*., 2012b). BLASTP v2.2.30 was used to detect the homologous gene pairs within the sesame genome (*E*‐value cutoff ≤ 1e‐20) (Altschul *et al*., 1997). Using the location of these target genes on chromosomes of sesame, the adjacent genes were considered a result of TD event.

### Phylogeny and divergence time inference

Using the gene clusters from *S. indicum* var. Zhongzhi13, Yuzhi11 and Swetha, *S. indicum* cv. Baizhima and Mishuozhima, *U. gibba* PLAZA_v4, *S. lycopersicum* SL2.50, *S. tuberosum* SolTub_3.0, *V. vinifera* IGGP_12x, *A. thaliana* TAIR10, *Z. mays* AGPv4 and *O. sativa* IRGSP‐1.0, the coding sequences (CDS) of 1010 single‐copy gene families within 12 plant species were used to construct a concatenated sequence alignment, which contained 1 089 576 common DNA sites. After removing unreliable sites by Gblock v0.91b (Talavera and Castresana, 2007), 518 199 common DNA sites were used to construct a phylogenetic tree using PhyML (Guindon *et al*., 2010) software with GTR+ Γ model for phylogenetic analysis of 12 plant species.

Using topology of phylogeny of 12 plant species and 30 637 fourfold degenerative sites from above alignments of single‐copy gene families, divergence times were estimated by PAML (v4.4b) (Yang, 1997) package with ‘mcmctree’ program. The following constraints were used for time calibrations from Timetree (http://timetree.org/) (Kumar *et al*., 2017): (i) The *O. sativa* and *Z. mays* diverged from 40 to 53 MYA; (ii) The *V. vinifera* and Pentapetalae diverged from 110 to 124 MYA; (iii) The monocots and dicots diverged from 148 to 173 MYA.

### Positive selected and fast‐evolving genes

To perform the analysis of positive selection, we obtained a new gene set of orthologous gene pairs using five sesame varieties and five dicots including *U. gibba* PLAZA_v4, *S. lycopersicum* SL2.50, *S. tuberosum* SolTub_3.0, *V. vinifera* IGGP_12x, *A. thaliana* TAIR10. Using BLAST v2.2.30 search with *E*‐value cutoff = <1e‐05, we identified 7956 orthologous gene pairs with reciprocal best hits among ten species (Altschul *et al*., 1997). We then used GUIDANCE v1.41 (Penn *et al*., 2010) to perform multiple sequence alignments with the parameters of seqType = codon, seqCutoff = 0.3, and msaProgram = muscle.

We estimated the d*N*/d*S* ratio (ω) using PAML v4.4b (Yang, 1997) with the coding sequence alignments above to detect the selection pressure on corresponding gene pairs. Firstly, we estimated the ω values using branch models (mode = 2 and NSsite = 0; Zhao *et al*., 2010) across the topology of ten plant species based on the tree: [(((((((*S. indicum* var. Zhongzhi13, *S. indicum* var. Yuzhi11), *S. indicum* cv. Baizhima), *S. indicum* cv. Mishuozhima), *S. indicum* var. Swetha) #1, *U. gibba*), (*S. tuberosum*,* S. lycopersicum*)), *V. vinifera*), *A. thaliana*] with the following parameters: Codonfreq = 2; kappa = 2.5; initial omega = 0.2. The three different hypotheses were used: (i) H0 hypothesis, all branches have the identical ω value; (ii) H1 hypothesis, the branch of five sesame varieties has a single ω value whereas the other branches have another identical ω value; (iii) H2 hypothesis, all branches have different ω values. We performed a LRT (likelihood‐ratio test) to select target genes whose likelihood values of H1 were significantly larger (adjusted LRT *P*‐value of < 0.01) than that of H0 and likelihood values of H2 were not significantly larger than that of H1. The genes which had larger ω values in sesame than other branches were considered to be fast evolving [rate (FDR)‐corrected *P*‐values (<0.01)].

We detected the genes with positively selected sites in the five sesame varieties using the branch‐site models (model = 2 and NSsite = 2). For null hypothesis, we used the parameters of ‘fix_omega’ = 1 and ‘omega’ = 1, but ‘fix_omega’ = 0 and ‘omega’ = 1.5 for the alternative hypothesis with tree: [(((((((*S. indicum* var. Zhongzhi13, *S. indicum* var. Yuzhi11), *S. indicum* cv. Baizhima), *S. indicum* cv. Mishuozhima), *S. indicum* var. Swetha) #1, *U. gibba*), (*S. tuberosum*,* S. lycopersicum*)), *V. vinifera*), *A. thaliana*]. We used an FDR‐corrected LRT with *P*‐value (adjusted LRT *P*‐value) cutoff = <0.01 to identify genes positively selected sites in sesame.

### Availability of supporting data

The original and improved assembly genome sequences, gene sets and function annotation of *S. indicum* var. Zhongzhi13, *S. indicum* var. Yuzhi11, *S. indicum* var. Swetha, *S. indicum* cv. Baizhima and *S. indicum* cv. Mishuozhima are available at http://www.sesame-bioinfo.org/pan-genome.

## Competing interests

The authors declare that they have no competing interests.

## Funding

This work was supported by the Natural Science Foundation of Hubei Province (grant number: 2017CFB612), the Agricultural Science and Technology Innovation Project of the Chinese Academy of Agricultural Sciences (CAAS‐ASTIP‐2013‐OCRI), Fundamental Research Funds for Central Non‐profit Scientific Institution, and China Agriculture Research System (CARS‐14).

## Authors’ contributions

JYY and XRZ contributed to the design of the research. DDF and JFC participated in the genome analysis. YXZ, JY, YJZ and LHW participated in the study design. AAG, KD and XW participated in finalization of the manuscript. KL participated in the analysis of lipid metabolism. DHL and RZ prepared materials. All authors read and approved the final manuscript.

## Supporting information


**Figure S1** Geographical distribution of the five sesame varieties in China and India.

**Figure S2** Venn diagram of the new and old gene sets in Zhongzhi13 genome.

**Figure S3** Comparison of different types of protein‐coding genes from core and dispensable genomes in sesame.

**Table S1** Statistics of the length of different chromosomes in five sesame genomes.

**Table S2** Detail information of predicted protein‐coding genes among five sesame varieties.

**Table S3** Statistics of gene families among 12 plant species.

**Data S1** The function annotation of new predicted protein‐coding genes in Zhongzhi13.

**Data S2** The collinear analysis of five sesame varieties compared to grape genome.

**Data S3** The tandem duplicated genes among five sesame varieties.

**Data S4** The specific genes in sesame modern cultivars and landraces.

**Data S5** The specific genes in Chinese and Indian modern cultivars.

**Data S6** KEGG analysis of expended and contracted gene families in five sesame varieties.

**Data S7** The function annotaion of positively selected and fast‐evolving genes in sesame.
