# Supplementary figures and images for: Insight into the evolution and functional characteristics of the pan‐genome assembly from sesame landraces and modern cultivars
Source: Plant Biotechnol J. 2018 Dec 8;17(5):881–92. doi: 10.1111/pbi.13022 (PMC6587448; doi:10.1111/pbi.13022)

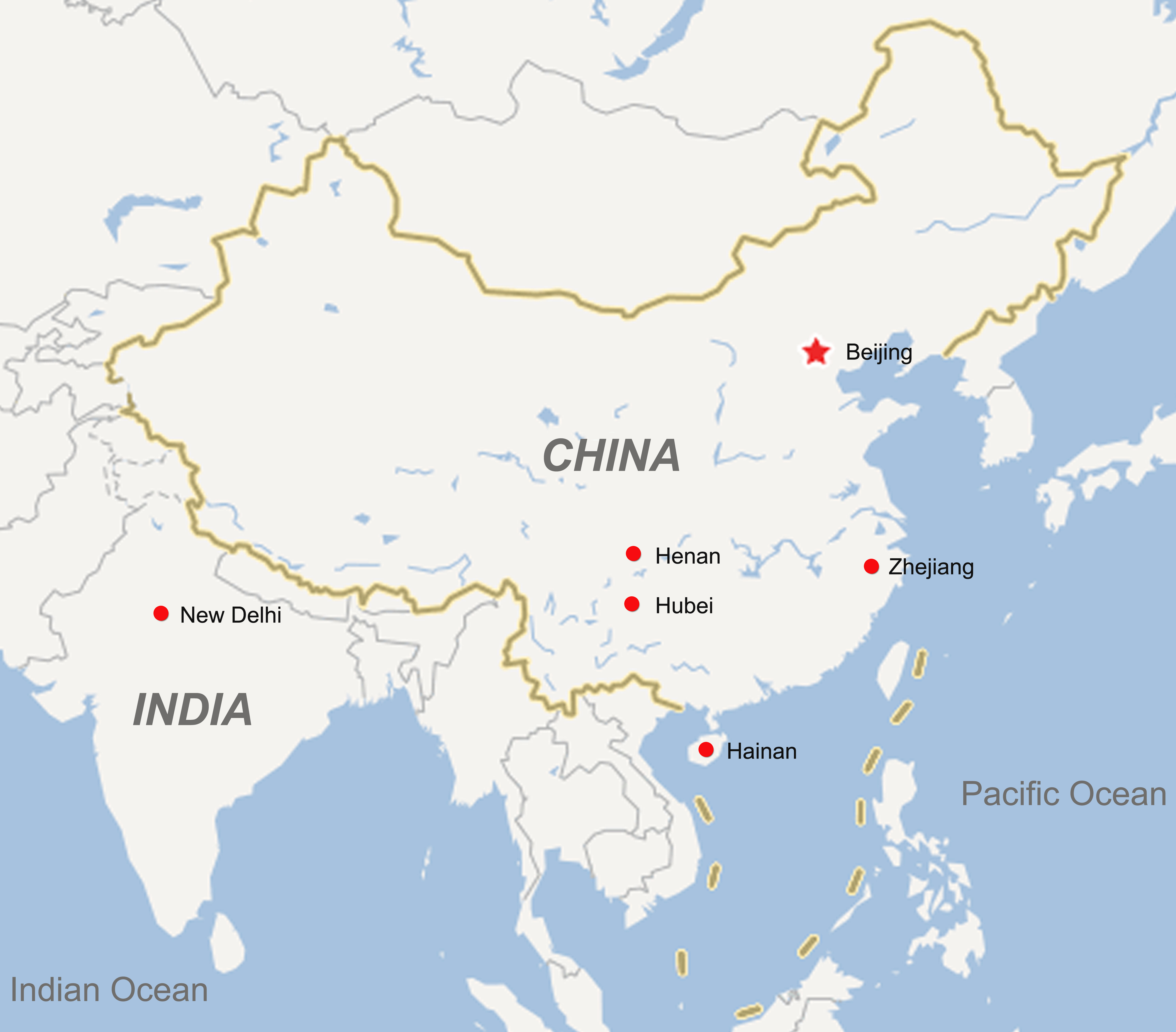

Supplement: Supplementary file 1 — Figure S1 Geographical distribution of the five sesame varieties in China and India. [file PBI-17-881-s006.tif]

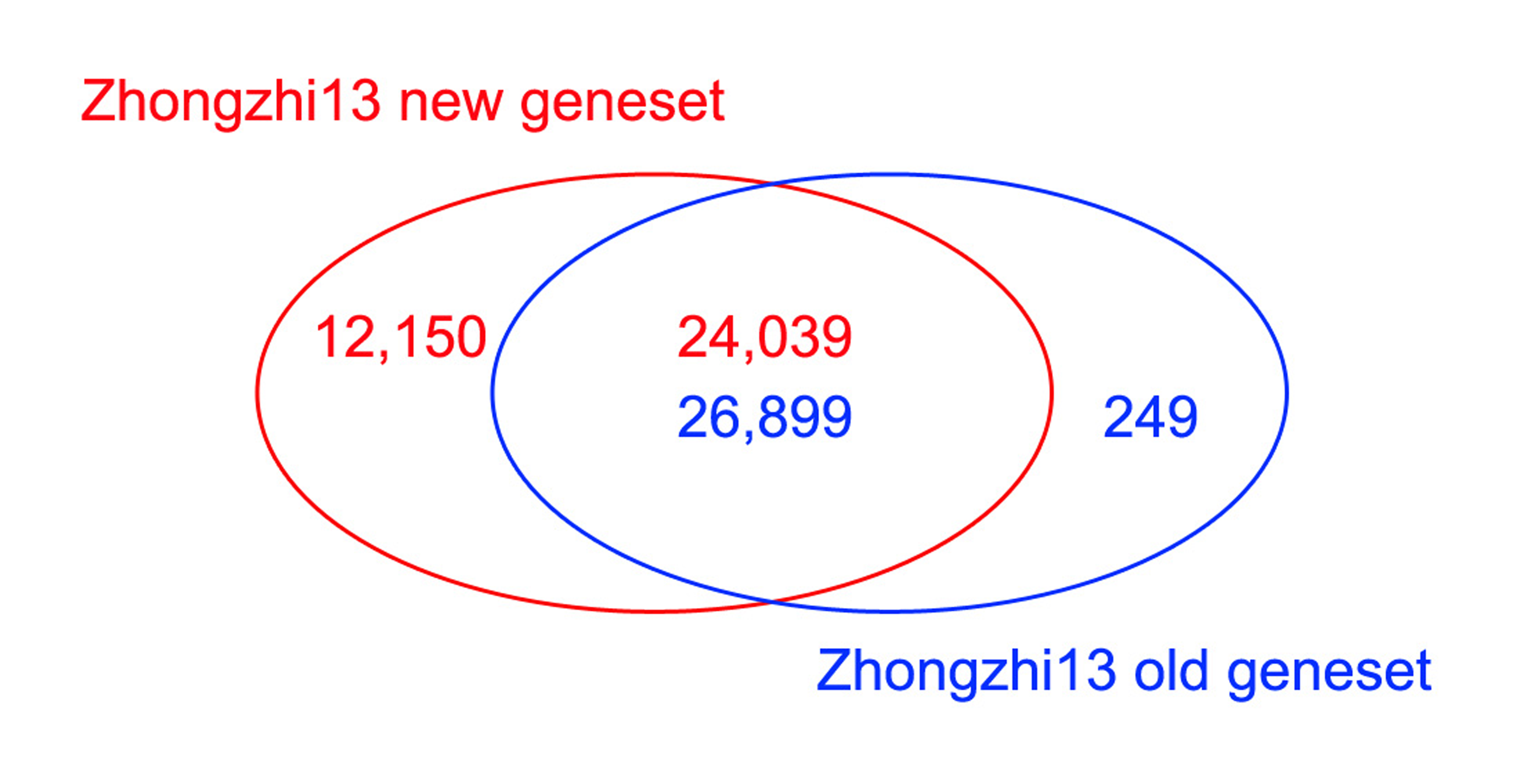

Supplement: Supplementary file 2 — Figure S2 Venn diagram of the new and old gene sets in Zhongzhi13 genome. [file PBI-17-881-s011.tif]

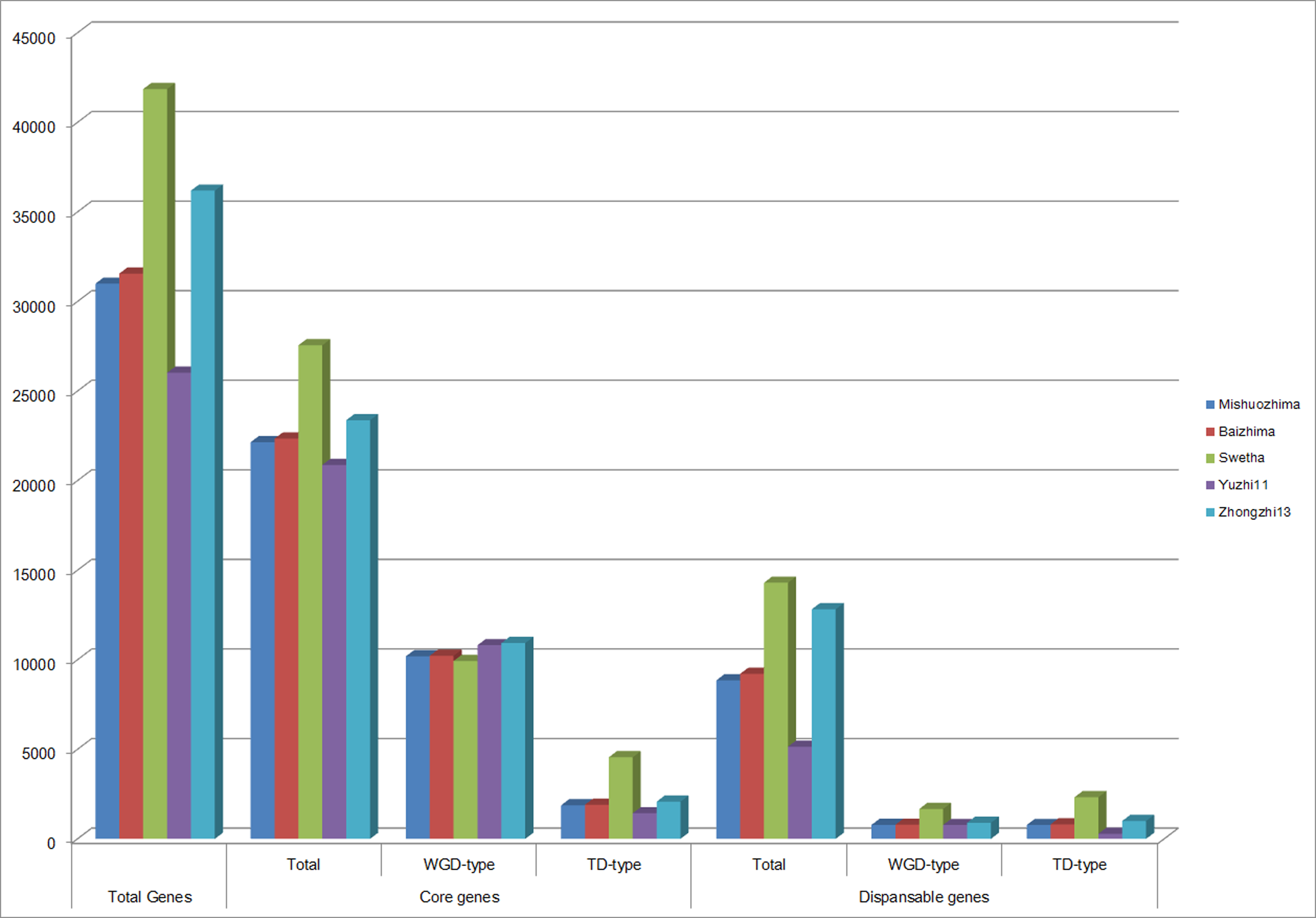

Supplement: Supplementary file 3 — Figure S3 Comparison of different types of protein‐coding genes from core and dispensable genomes in sesame. [file PBI-17-881-s010.tif]
