# Supplementary material for: Insight into the evolution and functional characteristics of the pan‐genome assembly from sesame landraces and modern cultivars
Source: Plant Biotechnol J. 2018 Dec 8;17(5):881–92. doi: 10.1111/pbi.13022 (PMC6587448; doi:10.1111/pbi.13022)
Supplement: Supplementary file 4 — Table S1 Statistics of the length of different chromosomes in five sesame genomes. [file PBI-17-881-s012.pdf]

Table S1. Length of different chromosomes among five sesame varieties.

Chromosomes	Zhongzhi13	Yuzhi11	Swetha	Baizhima	Mishuozhima
chr1	20,257,639	12,144,622	20,388,621	16,884,392	17,370,318
chr2	18,415,740	12,915,212	23,333,770	15,525,223	16,328,919
chr3	25,850,335	17,785,867	30,392,377	21,718,316	21,652,934
chr4	20,582,917	12,372,216	24,785,314	17,303,766	17,134,316
chr5	16,584,689	10,031,574	18,293,400	13,863,986	13,937,600
chr6	25,967,286	18,405,807	31,370,383	22,640,701	22,313,205
chr7	16,756,707	10,398,139	17,970,893	13,032,758	13,399,501
chr8	26,180,356	14,606,885	29,906,901	21,018,680	20,127,410
chr9	22,847,643	17,771,763	29,228,532	19,605,500	19,600,886
chr10	19,487,738	13,024,305	23,861,055	16,359,355	15,737,590
chr11	14,047,238	10,685,523	16,560,068	12,110,835	11,541,233
chr12	16,275,532	11,343,251	24,078,102	13,939,448	14,404,523
chr13	16,472,772	10,087,436	19,120,267	13,821,448	14,070,408
**Total Bases (chromosomes)**	259,726,592	171,572,600	309,289,683	217,824,408	217,618,843
**Ratio of anchored on chromosomes**	97.51%	81.52%	91.14%	81.87%	85.95%
