# Supplementary material for: Insight into the evolution and functional characteristics of the pan‐genome assembly from sesame landraces and modern cultivars
Source: Plant Biotechnol J. 2018 Dec 8;17(5):881–92. doi: 10.1111/pbi.13022 (PMC6587448; doi:10.1111/pbi.13022)
Supplement: Supplementary file 5 — Table S2 Detail information of predicted protein‐coding genes among five sesame varieties. [file PBI-17-881-s001.pdf]

Table S2. Detail information of predicted protein-coding genes among five sesame varieties.

Species	Gene number	%good orf	Start code	Stop code	Single exon gene	% single exon gene	Average gene length (bp)	Average CDS length (bp)	Average exons per gene	Average exon length (bp)	Average intron length (bp)
Zhongzhi13	36,189	90.73%	94.33%	93.53%	6,667	19.67%	3,718	1,259	5.03	250	609
Yuzhi11	26,022	91.51%	94.50%	94.76%	4,908	18.86%	3,623	1,300	5.49	237	516
Swetha	41,859	90.69%	94.60%	93.12%	6,418	15.33%	4,032	1,225	5.4	227	636
Baizhima	31,558	90.79%	94.35%	93.70%	6,217	19.70%	3,673	1,266	5.15	246	579
Mishuozhima	30,995	90.87%	94.42%	93.78%	6,067	19.57%	3,700	1,265	5.19	244	579
