# Supplementary material for: Insight into the evolution and functional characteristics of the pan‐genome assembly from sesame landraces and modern cultivars
Source: Plant Biotechnol J. 2018 Dec 8;17(5):881–92. doi: 10.1111/pbi.13022 (PMC6587448; doi:10.1111/pbi.13022)
Supplement: Supplementary file 6 — Table S3 Statistics of gene families among 12 plant species. [file PBI-17-881-s002.pdf]

Table S3. Statistics of gene families among 12 plant species.

Species	Genes number	Genes in families	Unclustered genes	Family number	Unique families	Average genes per family
**Baizhima**	31,558	30,079	1,479	22,837	11	1.32
**Mishuozhima**	30,995	29,678	1,317	22,660	8	1.31
**Swetha**	41,859	33,621	8,238	20,662	185	1.63
**Yuzhi11**	26,022	24,907	1,115	19,384	8	1.28
**Zhongzhi13**	36,189	32,448	3,741	23,541	155	1.38
Utricularia gibba	25,930	18,227	7,703	10,640	551	1.71
Solanum lycopersicum	33,785	25,887	7,898	16,561	504	1.56
Solanum tuberosum	39,021	31,796	7,225	16,238	800	1.96
Vitis vinifera	29,927	22,403	7,524	13,417	778	1.67
Arabidopsis thaliana	27,628	23,649	3,979	12,835	866	1.84
Zea mays	39,498	32,527	6,971	15,880	1,820	2.05
Oryza sativa	35,679	24,645	11,034	15,365	1,326	1.6
